# Nanofiltration Membranes Containing a Metal–Polyphenol Network Layer: Using Casting Solution pH as a Tool to Tailor the Separation Performance

**DOI:** 10.1021/acsomega.4c04804

**Published:** 2024-11-08

**Authors:** Hluf Hailu Kinfu, Md. Mushfequr Rahman, Nicolás Cevallos-Cueva, Volker Abetz

**Affiliations:** a Institute of Membrane Research, Helmholtz-Zentrum Hereon, Max-Planck-Straße 1, 21502 Geesthacht, Germany; b Institute of Physical Chemistry, University of Hamburg, Martin-Luther-King-Platz 6, 20146 Hamburg, Germany

## Abstract

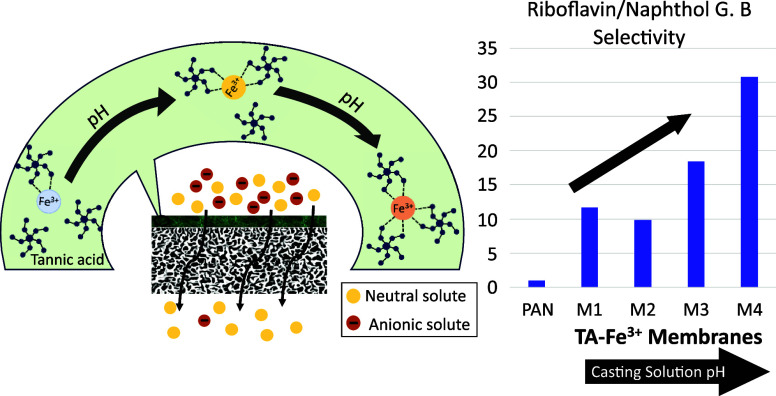

Thin-film composite (TFC) membranes containing metal–polyphenol network (MPN) selective layers were fabricated using a supramolecular self-assembly between tannic acid (TA) and ferric ion (Fe^3+^). The TA-Fe^3+^ thin film was coated on a porous polyacrylonitrile support using aqueous solutions of TA and FeCl_3_ via a layer-by-layer deposition technique. The pH of the TA solution was used as a tool to alter the membrane characteristics. The surface porosity and water contact angle of the fabricated membranes gradually decreased as the pH of TA casting solutions was increased from 3 to 8.5 for both single-layered and double-layered TA-Fe^3+^ TFC membranes. This allowed us to tune the water permeance and the retentions of water-soluble neutral and anionic molecules by the MPN membranes by varying the pH of the casting solution. It has been shown that the water permeance decreased from 184 to 156 L·m^–2^·h^–1^·bar^–1^ for single TA-Fe^3+^ layer coated membranes when the pH was increased from 3 to 8.5, while it declined from 51 to 17 for the double TA-Fe^3+^ layer. Anionic solutes in aqueous solutions were highly retained compared to neutral components as the TFC membranes had a negative surface charge. Retentions of 95 and 90% were achieved for naphthol green B and orange II dyes by a double-layered M4 membrane fabricated at pH 8.5, while only 13% retention was found for the neutral riboflavin. The neutral dye riboflavin permeated 30.8 times higher than the anionic dye naphthol green B during a mixed dye filtration test through the TFC membrane prepared by using a TA solution of pH 8.5. To the best of our knowledge, this is the highest selectivity of a neutral/anionic dye pair so far reported for a TFC membrane having an MPN selective layer. Moreover, fouling tests have demonstrated that the MPN separation layers exhibit robust stability and adequate antifouling performance with a flux recovery ratio as high as 82%.

## 1 Introduction

Nature-inspired methods hold great potential for fabricating high-performance materials. Among them, mussel-inspired metal–polyphenol coordination is receiving high attention in engineering novel functional surfaces.^1−5^ Metal–phenolic networks (MPNs) are self-assembled supramolecular structures comprising Polyphenols coordinating metal ions. Polyphenols can contain various catechol and galloyl functionalities. These are considered polydentate ligands that can chelate a vast range of transition metal ions to form highly stable MPNs.^6,7^ The facile fabrication and biocompatibility of MPN coatings have increased their application for the synthesis of innovative and multifunctional materials.^8^ The coating can be applied on various substrates due to the strong adhesion properties of Polyphenols through their catechol and galloyl moieties containing hydrophobic, hydrophilic, and charge shifting regions.^4^ The polyphenol–metal ion complexation has been identified as an efficient and versatile platform of surface modification to engineer interfaces of novel functionalities.^4,8,9^

Bioinspired approaches toward membrane development have found a wide scope and renewed interest among scientists.^10^ In the past decade, the environmentally sustainable fabrication of mussel-inspired MPN films as the separation layers of thin-film composite (TFC) membranes has attracted considerable attention in membrane separation technology.^11−13^ TFC membranes composed of a thin selective layer on top of a microporous support are the industrial standard in reverse osmosis, forward osmosis, and nanofiltration. In a TFC membrane, the thin selective layer is responsible for the separation, while the porous support provides the mechanical strength.^14^ Interfacial polymerization, the customary fabrication process of the thin selective layer of TFC membranes, involves the use of toxic organic solvents. The membrane industry consumes a large amount of hazardous solvents.^15,16^ Substitution of these solvents with greener solvents, such as water, is thus crucial. As a result of the global challenges linked with environmental contamination, environmentally friendly, greener procedures in membrane technology have become more popular.

The strong adherent properties, alongside its low cost, fast complexation kinetics, and facile and green preparation process,^9^ make the tannic acid (TA)-based MPN a suitable candidate to fabricate the selective layer of a TFC membrane. TA is a nature-derived substance.^17^ Moreover, the U.S. Food and Drug Administration has recognized MPN components such as TA as safe materials.^1^ TA is the most studied polyphenol and has been demonstrated to participate in various types of bondings.^18^ TA is not only an excellent polyphenol that forms films via metal chelation^1^ but also soluble in water.^19,20^ The coordination between TA and metal ions can alleviate contemporary environmental regulations on the use of organic solvents in membrane processes. These factors have prompted considerable research into the fabrication of TFC membranes with TA–metal ion coordinated selective layers.

Separation of small organic molecules (having dimensions between 0.5 and 5 nm) from their mixtures has received a lot of attention in recent years. Emerging contaminants, particularly from pharmaceuticals and textile industries, are still a global concern.^21,22^ NF membranes are becoming a reliable and potential tool for micropollutant removal.^23^ MPN-based membranes have been investigated for various applications since their introduction over the past decade. For instance, Fan et al. fabricated antioxidant TA-Fe^3+^ loose nanofiltration membranes.^11^ Lee and colleagues reported a facile approach to preparing antifouling MPN membranes for heavy metal removal.^13^ Fang et al. fabricated a PES/Fe-TA membrane via integrating blending and interfacial coordination for enhanced dye/salt separation.^24^ Several studies on the application of MPN-based thin films for oil-in-water emulsion separation have also been reported.^25−27^ Moreover, these TFC membranes have been applied for the removal of trace organic contaminants^12^ and dye removal.^28,29^ However, the use of TFC membranes containing TA-Fe^3+^-based selective layers for the separation of organic solutes and micropollutants from each other remains to be explored. Beyond the applications reviewed above, the implementation of TA-Fe^3+^ TFC membranes with significantly high separation efficacy for diverse applications such as pollutant removal and recovery is vital. This necessitates the development and design of a TA-Fe^3+^ layer having the right pore size and layer properties to be able to separate such small molecules from each other. Hence, it is important to explore the parameters that control the formation of ligand–metal ion complexes while casting the TA-Fe^3+^ layer. The pH of the casting solution is one of the major factors governing the MPN layer formation.^30^ Polyphenol-metal stoichiometry is highly pH-dependent.^31^ During TA-Fe^3+^ TFC membrane fabrication through the LBL technique, the first step is TA adsorption on the porous support. The acidity or alkalinity of the TA solution can influence its interaction with the support substrate. Second, the coordination with metal ions can be affected by solution pH^1^ during the self-assembly throughout the alternate deposition of precursor solutions. This entails the need for the systematic investigation of casting solution pH on the morphology and separation performance of the MPN membranes.

In the present work, we intend to demonstrate the effect of the TA casting solution pH on the membrane morphology and performance in the separation of neutral and anionic molecules having molecular weights in the range of 200–1000 g/mol. We report the fabrication of TA-Fe^3+^ TFC membranes via a layer-by-layer (LBL) method to form the MPN active layer on a porous PAN membrane support.

## 2 Experimental Section

### 2.1 Materials

The polyacrylonitrile (PAN) ultrafiltration membrane was fabricated at Helmholtz-Zentrum Hereon. TA (1701.2 g/mol) was purchased from Sigma-Aldrich Chemie GmbH (Germany). FeCl_3_·6H_2_O was obtained from Alfa Aesar GmbH & Co. Hydrochloric acid (HCl, 37%) was obtained from Merck Biosciences GmbH, while sodium hydroxide (NaOH) was provided by Sigma-Aldrich. Riboflavin (RB0), orange II (OR-), and naphthol green B (NGB3-) were purchased from Sigma-Aldrich Chemie GmbH (Germany). Poly(ethylene glycol) (PEG) of 200, 400, 600, and 1000 g/mol average molecular weights were obtained from VWR International GmbH (Germany). All of the chemicals in this study were used as received without further purification.

### 2.2 Preparation of TA-Fe^3+^ Membranes

The TFC membranes were fabricated by depositing TA and ferric salt solution over a microporous PAN support via an LBL strategy. The TA (0.1176 mM) and FeCl_3_ (3.330 mM) solutions were prepared separately by dissolving in water. These optimized concentrations have been chosen based on our previous rigorous study on the effect of concentration on membrane performance such as water flux and retention properties.^32^ The PAN support was first wetted by immersing it in water for 3 h. Then, the prewetted support was fixed between a glass plate and a PTFE frame. Each TA-Fe^3+^ layer was formed by exposing the top surface of the PAN membrane to a solution of TA for 4 min, followed by rinsing with pure water. Here, TA is adsorbed to the surface of the substrate. Next, the TA-adsorbed support membrane is exposed to a solution containing Fe^3+^ for the same period, leading to polyphenol–metal ion coordination, followed again by rinsing with pure water. In this way, membranes coated with one layer of TA-Fe^3+^ were fabricated. The pH of the aqueous solution in which TA was dissolved was varied between 3 and 8.5 to fabricate three membranes containing a single TA-Fe^3+^ layer.

The process was repeated again to obtain double-layered TA-Fe^3+^ TFC membrane through the LBL technique. The parameters used for coating the TA-Fe^3+^ layers on the PAN support are listed in Table 1.

**Table 1:** Membrane Separation Layer Fabrication Parameters for Single-Layered and Double-Layered TA-Fe^3+^ Thin Films of a Self-Assembled Network Deposited over the PAN Support with Their Respective Measured Pure Water Permeance (PWP)^a^

TA solution pH				
membrane code	first layer	second layer	number of TA-Fe^3+^ layers deposited	PWP in L·m^–2^·h^–1^·bar^–1^
S1	3		1	184
S2	5		1	167
S3	8.5		1	156
M1	3	3	2	51
M2	5	5	2	24
M3	5.8	5.8	2	21
M4	8.5	8.5	2	17
M5	3	5	2	45
M6	3	5.8	2	32
M7	3	8.5	2	26
M8	8.5	3	2	41
M9	8.5	5	2	26
M10	8.5	5.8	2	21
M11	5	8.5	2	34
M12	5	3	2	48
M13	5.8	3	2	46

a Casting solutions contain 0.1176 mM TA and 3.330 mM FeCl_3_, and the assembly time was 4 min.

### 2.3 Membrane Characterization

Fourier transform infrared (FTIR) spectra of the membranes were measured with a Bruker Alpha (diamond-ATR unit) (Bruker, Karlsruhe, Germany). The spectra in the range of 400–4000 cm^–1^ were collected using 64 scans at a resolution of 4 cm^–1^. The background spectrum of the PAN support was also acquired prior to TA-Fe^3+^ active layer synthesis. The zeta potential of the membranes was determined using a SurPASS 3 electrokinetic analyzer (Anton Paar, Austria). All tests were performed at room temperature using a background electrolyte solution of 1 mM NaCl. The surface hydrophilicity of the fabricated membrane was assessed through measurements of the water contact angle (WCA). The WCA measurements were performed on a KRUESS Drop Shape Analysis System DSA 100 (FEI part of Thermo Fisher Scientific, Kawasaki, Japan) at room temperature. DI water of 3 μL was dropped on the top surface in the sessile drop mode. Scanning electron microscopy (SEM) (Merlin SEM, Zeiss, Germany) was used to characterize the morphologies of the membranes. Samples were vacuum-dried at 60 °C for 72 h and then coated with 1–1.5 nm Pt using a CCU-010 coating device (Safematic, Switzerland) prior to imaging. SEM images of the cross section and top surface were acquired using accelerating voltages of 1.5 and 3 kV, respectively, at a working distance between 3.4 and 3.6 mm.

### 2.4 Filtration Performance Test

The membrane separation performances were evaluated through filtration tests in the dead-end filtration mode. PWP and solute rejection experiments were conducted at 3 bar transmembrane pressure after compaction at 4 bar for at least 2 h.

The water flux and permeance of the membranes were evaluated according to the following equations:
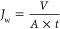
1
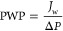
2where *J*_w_ (L·m^–2^·h^–1^) represents the water flux, while *V* (L) is the volume of the permeate, *A* (m^2^) is the effective filtration area, and *t* (h) is the operation time. PWP (L·m^–2^·h^–1^·bar^–1^) is the pure water permeance, and Δ*P* (bar) is the applied transmembrane pressure.

Rejection experiments were performed using a stirred test cell from Millipore (EMD Millipore XFUF07601). The feed solution was stirred at a rate of 350 rpm. Here, we determined the rejection performance of the TA-Fe^3+^ thin film using dye (0.1 mM) and PEG (1 g/L) solutions using 300 mL feed solutions. The molecular structure and the respective molecular weight and charge in a solution of the dyes used in this study are shown in Figure 8a. Moreover, mixed solute retention tests were performed using a 1:1 molar mixture of dye solutions of 0.1 mM concentration. Salt retention performances of the fabricated membranes were also examined with 1 g/L feed solutions of NaCl and a NaCl–Na_2_SO_4_ mixture. The mixed salt solution was prepared at a chloride-to-sulfate molar ratio of 1:1. Solute rejections were calculated using eq 3.^33^
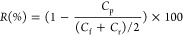
3where *R* is solute retention and *C*_p_, *C*_f_, and *C*_r_ are the solute concentrations in the permeate, feed, and retentate solutions in mg·L^–1^, respectively. Concentrations of the feed were determined before the test, and the concentrations of the permeate and retentate were determined after running the test. Three *C*_p_ and *C*_r_ samples were collected and averaged, each collected after 10 mL of permeate had passed. For dye filtration, the concentrations of the different samples were analyzed using a UV–vis spectrophotometer (GENESYS 10S, Thermo Scientific). However, PEG samples were analyzed with gel permeation chromatography (GPC) (VWR-Hitachi 2130 pump, Hitachi, Darmstadt, Germany). Ion chromatography (Dionex ICS600, Thermofischer Scientific Inc., USA) was used to analyze the concentrations of the ions during salt rejection measurements.

Membrane selectivity toward two solutes is computed using
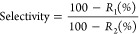
4

Here, *R*_1_ and *R*_2_ are rejections of solutes 1 and 2, respectively.

### 2.5 Assessment of Membrane Antifouling Property

The antifouling property of the MPN TFC membranes was evaluated with a dynamic filtration fouling test to determine the flux decline and flux recovery ratio (FRR). Humic acid solution (100 mgL^–1^) was used as a pollutant solution. The overall strategy involved three steps. First, the initial pure water flux (*J*_1_) of the membranes was measured for 1 h. Next, the feed solution was replaced by the humic acid solution, and the foulant solution flux (*J*_f_) was measured for 1.5 h. Then, the membrane was rinsed with pure water before being reinserted back into the filtration system to measure a new pure water flux (*J*_2_). This process is repeated again to achieve a two-cycle foulant solution filtration test. The FRR is then calculated according to the following equation:
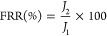
5

## 3 Results and Discussion

### 3.1 Membrane Morphology and Water Permeance

TA bears several −OH groups. The mechanism of coordination involves the release of two hydrogen ions into solution by TA to give two electrons to metal ions, thus leading to chelate formation.^34^ The complexation between TA and Fe^3+^ changes with solution acidity or alkalinity. As the dissociation of TA is pH-dependent,^35^ we investigated and characterized the effect of TA solution pH on TFC membrane fabrication and performance. The pH of the TA aqueous solutions was varied in the range of 3–8.5 to coat the selective layer of the TFC membranes on a microporous PAN support. At high pH, the phenolic functions of TA oxidize to quinone groups, which do not coordinate with Fe^3+^.^36^ Therefore, we have limited the study to a maximum pH value of 8.5 in this work, where no quinones have yet been formed. The pH of the FeCl_3_ solution was kept constant while coating the selective layers. Table 1 shows the PWP of the fabricated TFC membranes containing both single (S1–S3) and double (M1–M13) TA-Fe^3+^ layers. MPN coatings of one or two layers were investigated as further deposition of TA-Fe^3+^ layers resulted in the formation of highly dense films with a significantly reduced water flux.^32^ The surface chemical groups of the MPN coating and the microporous support were analyzed through FTIR analysis (Figure 2). Similar FTIR results for all of the fabricated membranes can be found in Figure S1 of the Supporting Information. Peaks at 2937 and 2242 cm^–1^ belong to the CH_2_ stretching vibration and the C≡N stretching vibration of the PAN support layer, respectively. The band at 1453 cm^–1^ corresponds to the bending of the C–H bond. FTIR characteristic peaks of different functional groups unique to TA were also detected. The peaks at 1202 and 1575 cm^–1^ are ascribed to the C–O stretching vibration and the C=C stretching vibration from the aromatic groups of TA, respectively. The absorption peak attributed to C=O stretching vibration from the ester groups of TA appears at 1713 cm^–1^, while the broad band at 3100–3700 cm^–1^ that belongs to the stretching vibration of O–H groups was enhanced in the newly synthesized double-layered membranes due to their phenolic nature. The occurrence of the above-mentioned new bands confirms the deposition of TA-Fe^3+^ films.

The PWP of the pristine PAN support was ∼266 L·m^–2^·h^–1^·bar^–1^. The TA-Fe^3+^ layer coated membranes allowed significantly lower water permeances than that of the pristine PAN support. The water permeance of the membranes containing a single TA-Fe^3+^ layer (i.e., S1, S2, and S3) declined from 184 to 156 L·m^–2^·h^–1^·bar^–1^ (Table 1 and Supporting Information Figure S2) as the pH of the TA casting solution increased from 3 to 8.5. Figure 3 shows the surface and cross-section SEM micrographs of the PAN supports, S1, S2, and S3. Compared to PAN, the surfaces of S1, S2, and S3 showed a notable change in the porous structure due to the deposition of the MPN layer (Figure 3). The PAN support membrane showed a highly porous top surface structure, while the TA-Fe^3+^-coated membranes displayed a decrease in the number and size of surface pores. The cross-section images of all membranes showed a sponge-like structure. No substantial variations in the cross-sectional morphology between the PAN support and the TFC membranes were observed. It shows the formation of a thin TA-Fe^3+^ layer on the top surface without deep penetration into the pores of the PAN support layer.

M1–M4 are double TA-Fe^3+^ layered TFC membranes in which both layers were synthesized at the same pH. The PWP of the TA-Fe^3+^ membrane synthesized at pH 3 (M1) was ∼51.5 L·m^–2^·h^–1^·bar^–1^. A sharp decline in PWP is displayed when the pH of the TA casting solution is increased from 3 to 5. The PWP of TA-Fe^3+^ membranes prepared at pH 5, pH 5.8, and pH 8.5 are 24, 21, and 17 L·m^–2^·h^–1^·bar^–1^, respectively. These results are in line with the observed SEM morphology (Figure 4a–d), which shows that the surface porosity of the assembled film decreases due to the enhanced coordination of neighboring TA-Fe complexes at high pH.

**Figure 1:**
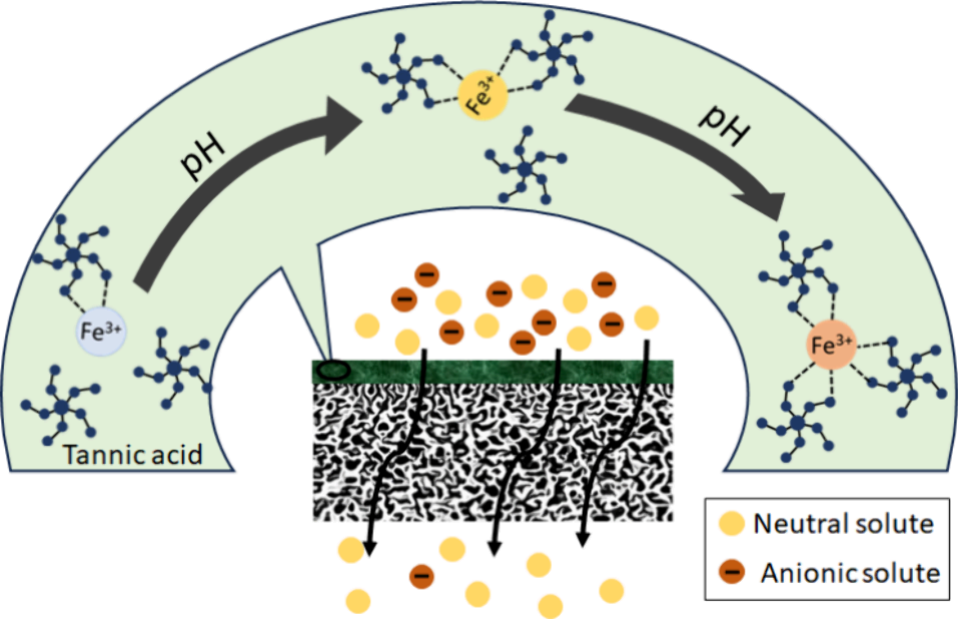
Schematic illustration of the TA-Fe^3+^ selective layer coating over a PAN porous membrane. The illustration shows the pH-dependent transition of the coordination between TA and Fe^3+^ ions among mono-, bis-, and tris-complex states, and the resulting thin film’s selective permeation behavior toward organic solutes.

At specified concentrations, assembly times, and ionic strengths, the aqueous solution pH controls the polyphenol–metal ion complexation nature as illustrated in Figure 1. The metal–polyphenol complexes comprise various stoichiometries (i.e., mono-, bis-, and tris-complex) as modulated by pH. The metal chelation behavior of catechol and galloyl ligands demonstrates that acidity or alkalinity of the solution controls the ionization of TA.^37^ Essentially, several phenol groups are protonated at low pH. Phenol groups of TA only coordinate with a metal ion in the deprotonated state, i.e., when they are negatively charged. Therefore, only a small number of functional groups are available for coordination. This results in larger open pores between neighboring complexes. Hence, a low cross-linked network of the dominantly monocomplex coordination state is formed. Deprotonation of TA and the number of available metal binding sites increase with an increase in the pH of the aqueous solution. TA-Fe^3+^ coordination is predominantly in the bis-complex state in the solution pH range of 3–6.^1^ However, at pH 7 and higher, a dense highly cross-linked self-assembled film of the tris-complex state displaying low water permeance is synthesized. The tris-complex elucidates three TA molecules coordinated with one metal ion center.

**Figure 2:**
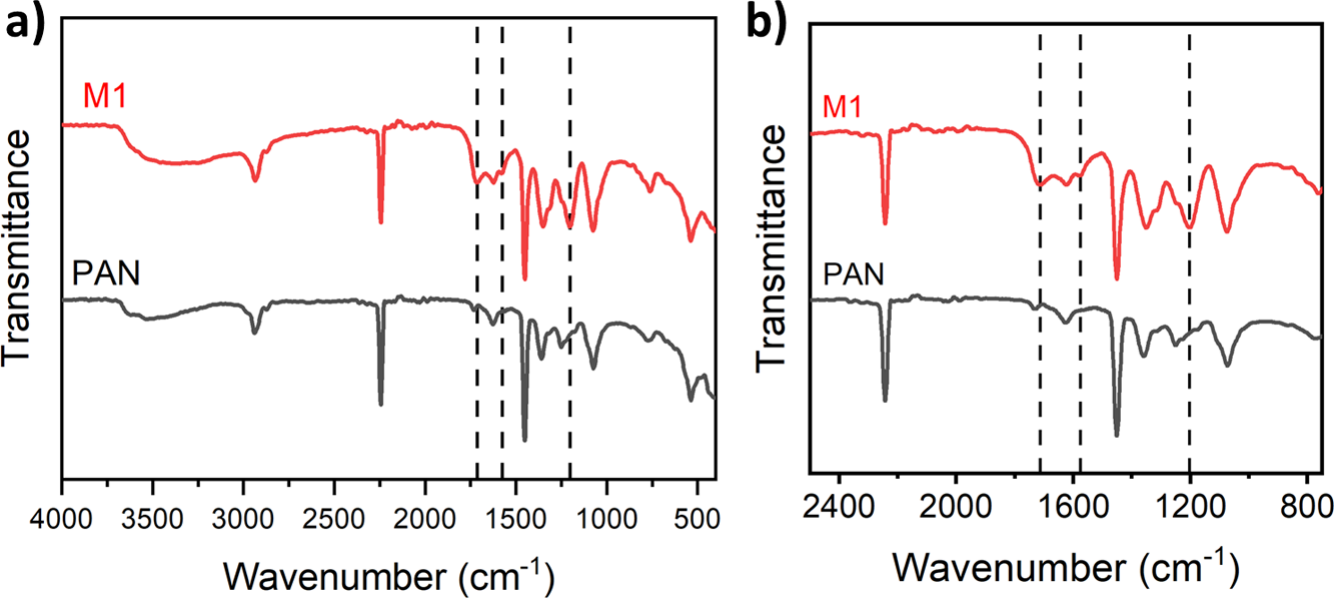
(a) FTIR spectra of PAN and a double TA-Fe^3+^ layered TFC membrane fabricated at pH 3 for both layers (M1), and (b) selected FTIR region illustrating the peaks specific to TA.

**Figure 3:**
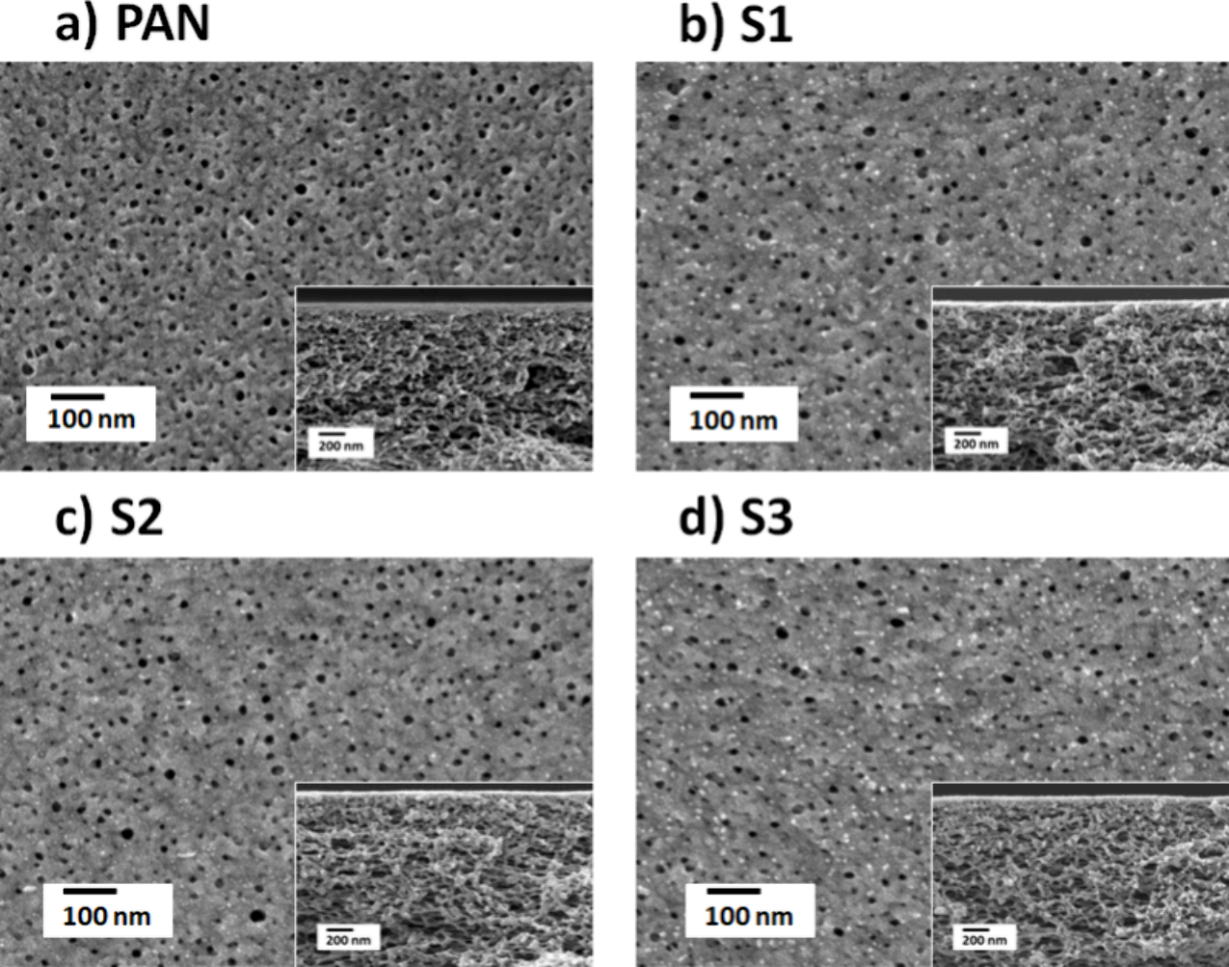
SEM images of single-layered TA-Fe^3+^ TFC membranes fabricated at different pHs: (a) pristine PAN support, (b) pH 3, (c) pH 5, and (d) pH 8.5. Insets show cross-sectional SEM images. The fabrication parameters can be found in Table 1.

**Figure 4:**
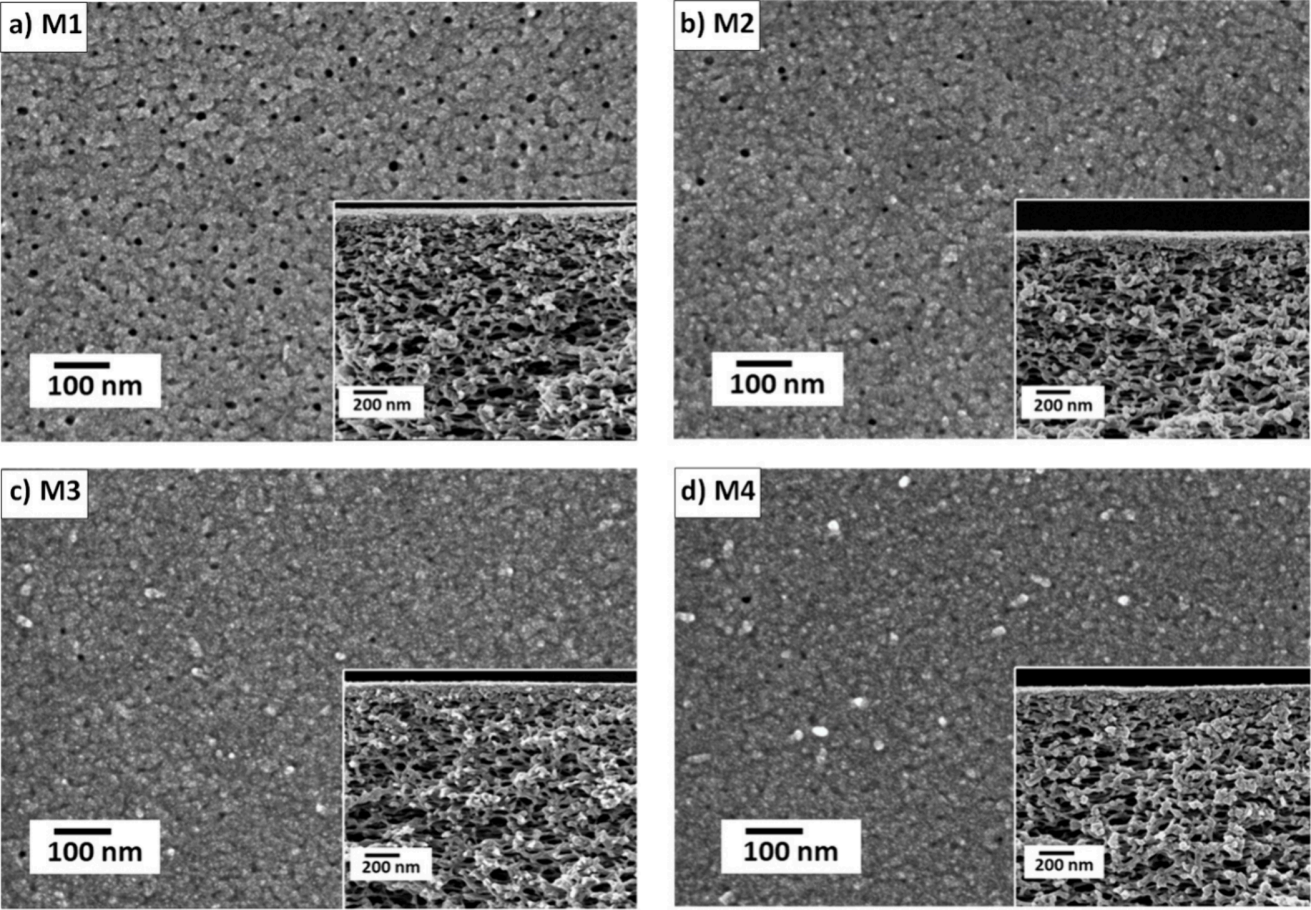
SEM images of double-layered MPN TFC membranes fabricated at the same pH for both TA-Fe^3+^ layers: (a) M1 at pH 3, (b) M2 at pH 5, (c) M3 at pH 5.8, and (d) M4 at pH 8.5. Insets show their cross-sectional SEM images. The fabrication parameters can be found in Table 1.

**Figure 5:**
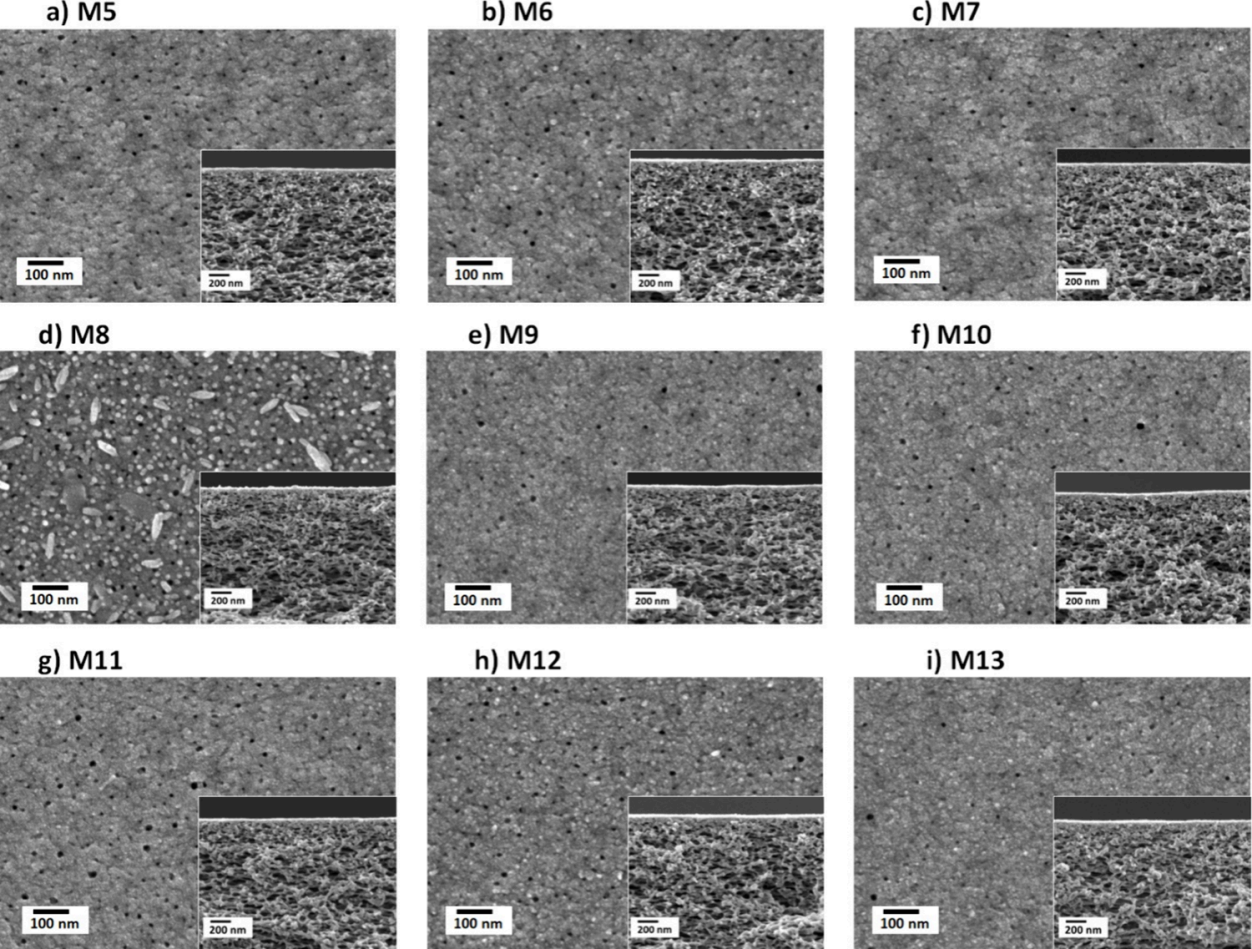
Surface and cross-sectional SEM images of double-layered TA-Fe^3+^ TFC membranes prepared at different pH conditions for each layer. The fabrication parameters can be found in Table 1.

In the cases of M5–M13, we have also explored the interplay between the PWP and casting conditions by the deposition of each TA-Fe^3+^ layer at a different pH (Table 1). The surface and cross-sectional SEM micrographs of these membranes are provided in Figure 5. Two sets of coating solutions were analyzed in this work. For M5, M6, and M7, the first MPN layer is deposited using a TA solution of pH 3, while for the second layers, TA solutions of pH 5, 5.8, and 8.5 were used. The PWP decreases with increasing the pH of the casting solution of the second layer. The PWP of M5 (pH 3 for the first layer and pH 5 for the second layer) was 45.5 L·m^–2^·h^–1^·bar^–1^, while it declined to 26 L·m^–2^·h^–1^·bar^–1^ for M7 (pH 3 for the first layer and pH 8.5 for the second layer). The fabrication of M8, M9, and M10 involves coating the first TA-Fe^3+^ layer using a TA solution of pH 8.5 and the second layers using TA solutions of pH 3, 5, and 5.8. The PWPs of M8, M9, and M10 are 41, 26, and 21 L·m^–2^·h^–1^·bar^–1^, respectively (Table 1 and the Supporting Information, Figure S2). The comprehensive investigation allows us to analyze the role of the first and second TA-Fe^3+^ layers in the resistance against mass transport through the membrane. Considering the PWP through the PAN support as the basis, for a TA casting solution of pH 3, the PWP dropped by 31% after coating the first TA-Fe^3+^ layer (i.e., in the case of S1), while it dropped by 81% after coating the second TA-Fe^3+^ layer (i.e., in case of M1). In the case of the pH 8.5 TA solution, the first and second coatings of the TA-Fe^3+^ layer led to 41 and 94% drops of the PWP, respectively. In the double TA-Fe^3+^ layered membranes, the second TA-Fe^3+^ layer plays the dominant role in determining the resistance against mass transport. However, an obvious difference of PWP was also observed for the membranes where the first TA-Fe^3+^ layers were coated using TA solutions of different pHs, while the second membrane was coated using the same TA solution. For example, the PWP through M8 is approximately 10 L·m^–2^·h^–1^·bar^–1^ lower than that of M1. The second TA-Fe^3+^ layer of both M1 and M8 is coated using the TA solution of pH 3. The difference in PWP in these membranes stems from the first TA-Fe^3+^ layer, which is coated using a TA solution of pH 3 and 8.5 for M1 and M8, respectively. Similarly, a 9 L·m^–2^·h^–1^·bar^–1^ difference in PWP is observed between M7 and M4 as the first TA-Fe^3+^ layers of these membranes are coated using TA solutions of pH 3 and 8.5, respectively, although the second layers for both membranes are coated using the TA solution of pH 8.5. Therefore, we also attempted to analyze the influence of reversing the pH of the TA casting solutions used to coat the first and second TA-Fe^3+^ layers of the TFC membranes. The first and second layers are reversed using TA casting solutions of pH 3 and 8.5 to coat the selective layers of M7 (first pH 3 and second pH 8.5) and M8 (first pH 8.5 and second pH 3). The PWP of M7 (26 L·m^–2^·h^–1^·bar^–1^) < M8 (41 L·m^–2^·h^–1^·bar^–1^). Similarly, the PWP of M6 (32 L·m^–2^·h^–1^·bar^–1^) < M13 (46 L·m^–2^·h^–1^·bar^–1^), which shows the influence of reversing the first and second TA-Fe^3+^ layers using TA casting solutions of pH 3 and 5.8. The PWP of M9 (26 L·m^–2^·h^–1^·bar^–1^) is slightly lower than that of M11 (34 L·m^–2^·h^–1^·bar^–1^). The influence of using TA casting solutions of pH 5 and 8.5 to reverse the first and second layer coatings on PWP can be observed from this pair. The PWP of M5 (45 L·m^–2^·h^–1^·bar^–1^) ≈ M12 (48 L·m^–2^·h^–1^·bar^–1^). This pair reversed the first and second TA-Fe^3+^ layers coated using the TA casting solutions of pH 3 and 5. In general, the average PWP of the double TA-Fe^3+^ layered membranes fabricated using TA solutions of higher pH for the first layer surpassed those for which lower pH TA solutions were used to coat the first layer. When the pHs of the TA casting solutions used to coat the first and second TA-Fe^3+^ layers are far away from each other (e.g., pH 3 and 8.5), the effect of reversing is also stronger. Overall, the results show that although the PWP of the TFC membranes can be tuned for the single TA-Fe^3+^ layered membranes coated using TA solutions (0.1176 mM) of different pHs, these membranes might be useful for ultrafiltration applications. This work aims to prepare nanofiltration membranes for the separation of small organic molecules (having MW between 200 and 1000 g/mol). The PWP values of the double-layered membranes varied between 21 and 51 L·m^–2^·h^–1^·bar^–1^, which is typical for loose nanofiltration membranes with large molecular weight cutoffs suitable for the separation of organic molecules.^38,39^ Hence, among the membranes listed in Table 1, only those containing a double TA-Fe^3+^ layer can be suitable for the targeted applications. For further investigations, we have selected M1, M2, M3, and M4 (i.e., the double TA-Fe^3+^ layered membranes), where both layers are coated using the TA solutions of the same pH.

### 3.2 WCA and Surface Zeta Potential

**Figure 6:**
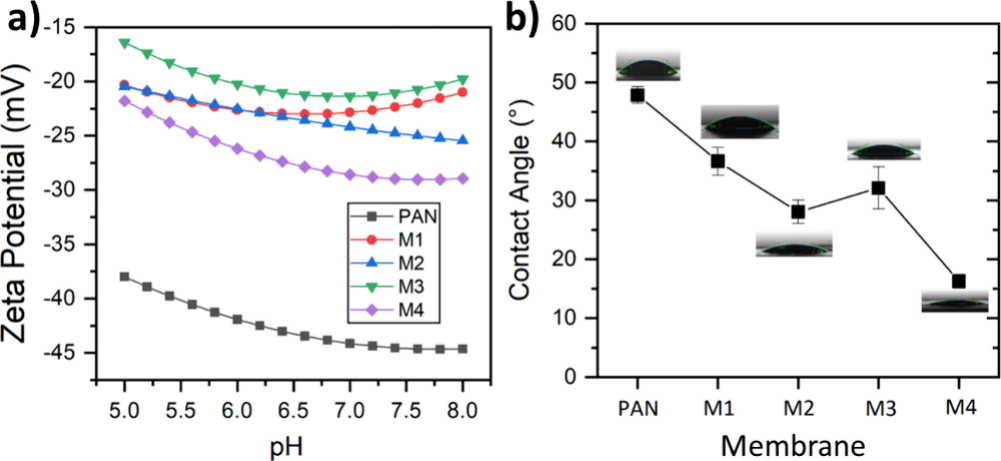
(a) Surface zeta potentials as a function of background electrolyte pH and (b) WCA of the membranes.

Mussel-inspired metal–polyphenol coatings have a reputation for producing stable and hydrophilic surfaces.^40,41^ WCA was used to examine the change in hydrophilicity before and after MPN layer coating. The wettability of the membranes prepared at various pH values as revealed by WCA is presented in Figure 6b. The pristine support showed a WCA of around 47 °, illustrating its relatively low hydrophilic property. The PAN water wettability is due to the high polarity of its backbone.^42^ After *in situ* fabrication of the TA-Fe^3+^ thin film, the WCA of the TFC membranes declined gradually. The widely accepted notion is that a reduced contact angle signifies increased surface energy, a stronger tendency of membrane wetting by water, and therefore enhanced hydrophilicity. An increase in the pH of the casting solution resulted in the fabrication of a comparatively hydrophilic surface. The WCA of the membrane M4 decreased to about 16 °. Time-dependent WCA from WCA tests is shown in Figure S4 of the Supporting Information. TA-based selective layers exhibit a very low WCA, indicating the fabrication of superhydrophilic membrane surfaces.^43^ The low contact angles of the TFC membranes were due to the hydrophilic nature of the TA-Fe^3+^ top layer containing hydroxyl functional groups in polyphenols. The functional groups of TA on the membrane surface can form hydrogen bonds with water molecules. The nanostructured protrusions on the surface of the membrane also contribute to the overall hydrophilicity.^44^ Enhancing the surface hydrophilicity is an effective approach for increasing the water permeance of membranes.^43^ Hence, the synthesis of an MPN selective layer with low WCA decreases the membrane surface transport resistances toward water molecules. Moreover, hydrophilic surfaces prevent membrane fouling by inducing the formation of a hydration layer at the membrane/water interface.^45^

Membrane performance is predominantly affected by the active layer characteristics, such as surface charge and pore size. Figure 6a shows the pH dependence of the membrane surface zeta potential curves. The results of the zeta potential demonstrated that the negative surface charge of the TFC membranes was lower than that of the PAN support. This weaker negative charge of the membrane surface after MPN coating has been reported before.^32,46^ However, all membranes exhibited a negative zeta potential in the pH range of 5–8. The charge of a membrane plays a crucial role in determining its ability to reject solutes during filtration processes.^47^ Surface charge has a strong influence on membrane separation performance owing to the Donnan effect. Therefore, the observed negative surface charges of the TA-Fe^3+^ layer would significantly impact the membrane rejection mechanism, enhancing the subsequent anionic solute rejection and selectivity. Moreover, the negative charge of the membrane surface enables the membrane to provide better antifouling performance.^48^ The observed electronegativity of the TFC membrane surface is attributed to the ionizable functional groups in the TA-containing self-assembled network.

### 3.3 Membrane Separation Performance

#### 3.3.1 PEG Retention

**Figure 7:**
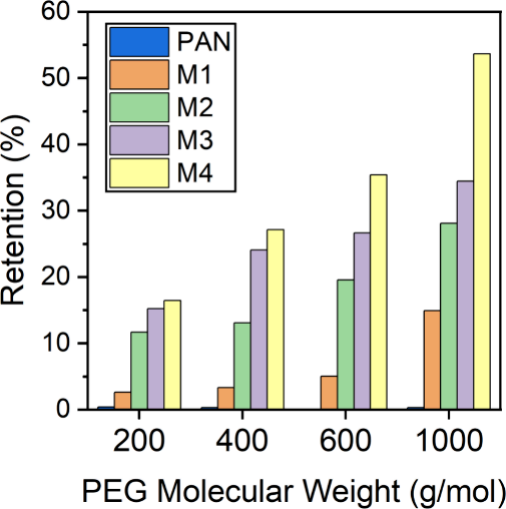
Neutral solute retention performance of double TA-Fe^3+^ layered membranes M1, M2, M3, and M4. Retention of 1 g/mol feed solution of PEG 200–1000 g/mol MW at 3 bar transmembrane pressure.

Figure 7 shows the retention of PEG 200–1000 g/mol by PAN support, M1, M2, M3, and M4. As PEG is a neutral molecule, the retention of PEG of different molecular weights portrays the size sieving ability of the membranes. Figure 7 shows that the PAN membrane (which is used as a support layer for the TFC membranes) does not significantly retain PEG 200–1000 g/mol. In other words, the pores of the PAN membranes are large enough to allow a complete permeation of neutral molecules as large as PEG 1000 g/mol. Hence, the retention behavior of M1, M2, M3, and M4 solely represents the size sieving property of the TA-Fe^3+^ selective layers of the TFC membranes due to the variation of pH of the TA solution between 3 and 8.5. Owing to the high surface porosity, M1 has the lowest retention of the PEG molecules among the four membranes. The retentions of PEG 200–600 g/mol by M1 are below 10%, while only that of PEG 1000 g/mol exceeds 10%. An increase of PEG retention by the membranes is observed when the pH of the TA casting solution rises. For all four studied PEG molecular weights, the retention decreased in the order of M4 > M3 > M2 > M1. It demonstrates that the size sieving ability of the TA-Fe^3+^ self-assembled thin selective layer can be controlled by varying the TA aqueous solution pH. Figure 7 also demonstrates that M1, M2, M3, and M4 allow a rather high permeation of PEG 200–1000 g/mol. Only the retention of PEG 1000 by M4 exceeds 50%. In every other case, the retention values are below 40%. In other words, in the absence of electrostatic interaction, the neutral solutes of 200–1000 g/mol can permeate rather easily through M1, M2, M3, and M4.

#### 3.3.2 Single Dye Retention

**Figure 8:**
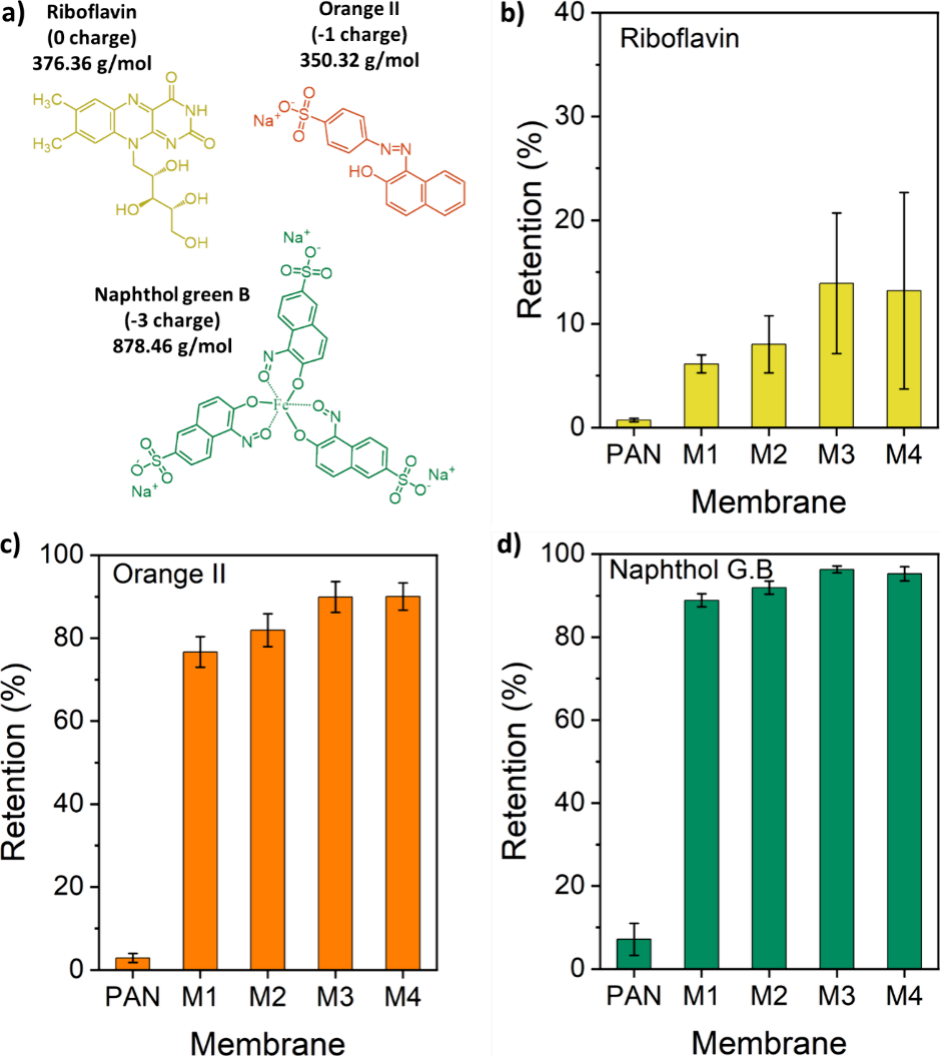
(a) Molecular structure of the organic solutes used for retention with their respective molecular weight and charge in aqueous solution, and single dye retention performance of the TA-Fe^3+^ membranes; (b) riboflavin, (c) orange II, and (d) naphthol green B retention.

Figure 8 shows the retention of three dyes from their respective aqueous solutions by M1, M2, M3, and M4. The molecular weights of orange II (OR-), riboflavin (RB0), and naphthol green B (NGB3-) are 350.32, 376.36, and 878.46 g/mol, respectively. Control experiments with the uncoated porous PAN membrane were performed first for analysis. Interestingly, this membrane exhibited low dye retention performances of only 1, 3, and 7% for riboflavin, orange II, and naphthol green B, respectively. The expected low rejections in the PAN likely stem from the enhanced convective transport of the dyes due to the large pore sizes of the support. The solutes encounter little to no hindrance in entering the membrane pore. However, the retention rates of the anionic dyes by the TFC membranes are significantly enhanced after MPN layer coating.

As the TA-Fe^3+^ membranes have a negative surface charge, OR- and NGB3- are highly rejected. For example, the retentions of OR- and NGB3- by M1 are around 79 and 89%, respectively. It is noteworthy that the retention of PEG 400 g/mol by M1 is only ∼3% (Figure 7). Although OR- has a molecular weight of 350.32 g/mol, owing to the electrostatic repulsion by the surface of the membrane, the retention of OR- by M1 is approximately 24 times higher than that of PEG 400 g/mol. As expected, the neutral RB0 was hardly retained by M1 (5%) although the molecular weights of RB0 and OR- are comparable. This reiterates the charge selectivity of the MPN layer due to the Donnan effects, while the influence of size sieving on solute retention is minimal. Membranes basically reject organic solute molecules primarily through a combination of size exclusion and charge repulsion mechanisms. The size exclusion mechanism relies on the relative size of the dye molecules and the membrane pores. However, the low retention of electroneutral solutes of PEG and riboflavin in correlation with the observed SEM images illustrates the small contribution of size sieving to the overall retention mechanism. Therefore, the high retentions of the anionic dyes are due to the electrostatic repulsion interaction between the negatively charged TA-Fe^3+^ membrane surface (Figure 6a) and the charges carried by the organic solutes in solution. This exclusion mechanism supplies the MPN membranes with a charge-selective property to effectively separate and remove dye molecules from the aqueous solutions. Membranes M2, M3, and M4 also show a similar behavior, i.e., a substantially higher retention of OR- compared to RB0. The retentions of OR- by M2, M3, and M4 membranes are in the range of 82–90% (Figure 8c), while those of RB0 are only between 8 and 14% (Figure 8b). The multivalent anionic NGB3- was highly rejected by the membranes. 92–96% of the NGB3- retentions were achieved by M2, M3, and M4. These membranes displayed notable uncharged dye/charged dye selectivities, as presented in Table S1 of the Supporting Information. The RB0/NGB3- ideal selectivities of the four membranes are between 8.5 and 18.2, while the RB0/OR- selectivities are between 4.5 and 8.7. Ideal selectivities were also observed for the OR-/NGB3- dye pair owing to the larger size and valency of NGB3- compared with that of OR-.

#### 3.3.3 Mixed Dye Retention

**Figure 9:**
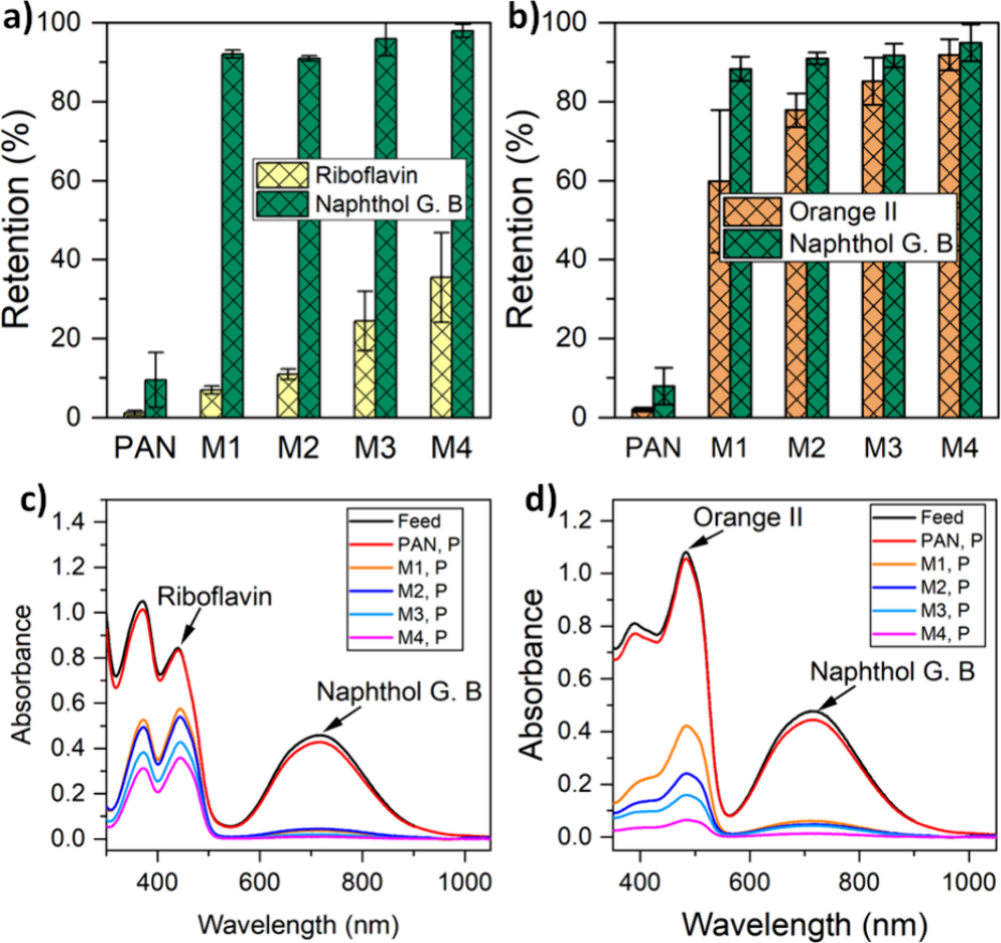
Mixed dye separation and selectivity of a (a) riboflavin and naphthol green B (RB0/NGB3-) mixture and (b) orange II and naphthol green B (OR-/NGB3-) mixture by double-layered membranes prepared at different pHs. M1, M2, M3, and M4 membranes were synthesized with aqueous solutions at pHs of 3, 5, 5.8, and 8.5, respectively. UV–vis spectra in (c) show riboflavin–naphthol green B separation with peaks at 444 and 715 nm, respectively, for the feed solution and the permeate samples of each membrane. The spectrum of the permeate (P) sample by the MPN membranes shows a disappearing peak attributed to naphthol green B, depicting effectual removal. (d) UV–vis spectra of the OR-/NGB3- mixture illustrating a decrease in the peaks of orange II (482 nm) and naphthol green B (715 nm) after filtration.

To investigate the separation performance of M1, M2, M3, and M4 in a mixed solute filtration, retention of a mixture of binary dyes was performed. A 1:1 molar mixture of riboflavin and naphthol green B (RB0/NGB3-) was used as an uncharged/charged dye mixture. Similarly, an equimolar mixture of negatively charged dyes, orange II and naphthol green B (OR-/NGB3-), was filtered by using the MPN membranes. During single dye retention, the membranes displayed a high riboflavin to naphthol green B ideal selectivity (Table S1 in the Supporting Information). For mixed dye solution filtration, 0.1 mM feed solutions were used. Figure 9 summarizes the results of the mixed dye solution tests. In both mixed dye experiments, the solutes easily permeated across the PAN control membrane, which showed no substantial dye retention (Figure 9). Almost no selectivities (∼1) for both RB0/NGB3- and OR-/NGB3- mixtures were obtained for the PAN membrane, and this further indicated that the support membrane has no significant influence on the retention or selectivity of the fabricated TFC membranes.

The MPN membranes can effectively separate the negatively charged NGB3- and neutral RB0 from the RB0/NGB3- mixture (Figure 9a). All membranes in the M1–M4 series displayed more than 90% retention toward naphthol green B. The M3 membrane that contains a double-layered TA-Fe^3+^ separation layer prepared at pH 5.8 can selectively remove 96% of the NGB3-, while the membrane synthesized at pH 8.5 (M4) removed 98% of the NGB3- from its mixture with RB0. As expected, the retentions of RB0 from the RB0/NGB3- mixture were significantly lower compared to those of NGB3-. However, a careful comparison of Figures 8b and 9a suggests that the retentions of RB0 from the feed solution containing the RB0/NGB3- mixture by the membranes were higher than those from the feed solution containing only RB0. M1, M2, M3, and M4 retained 7, 11, 25, and 35% of the RB0 from the feed solution containing the RB0/NGB3- mixture, respectively. Owing to the electrostatic exclusion, NGB3- molecules were constantly rejected from the membrane pores. Consequently, the permeation of anionic NGB3- lagged far behind the permeation of neutral RB0 through the membranes. The neutral RB0 had a substantially lower energy barrier compared to NGB3- to enter the pores of the membranes. In spite of that, RB0 had to compete with NGB3- for space to enter the pores. Such a competing effect resulted in a significant increase of RB0 retention when the feed solution contained the RB0/NGB3- mixture. The M4 membrane showed the best selective separation of neutral and anionic binary dye mixtures with a selectivity of 30.8, followed by the M3 membrane with 18.4 selectivity (Table 2). Thus, both M3 and M4 showed excellent performance in separating RB0 and NGB3- from their mixture. Both pore size and surface properties play a vital role during dye separation.^49^ The overall membrane rejection ability arises from the combined effect of steric and Donnan exclusion. Such RB0/NGB3- separation results indicate the potential to separate small-molecule-sized dye mixtures by manipulating the membrane structure and chemistry. The zeta potential experiments showed that M4, for example, has a stronger negative charge compared to that of M3. The water flux and PEG retention results proved that the effective pore size of M4 is smaller than that of M3. A combination of these two membrane properties leads to a superior RB0/NGB3- selectivity for M4 compared to M3. However, no significant difference was observed between the OR-/NGB3- selectivities of the M1–M4 membranes. The result of the OR-/NGB3- mixture separation by the fabricated membranes is shown in Figure 9b. It can only be noted that M1 showed the best OR-/NGB3- separation property. The selectivity of OR- over NGB3- was 3.4 (Table 2). The retentions of both anionic dyes increased with an increase in solution pH during film fabrication. The decrease in pore size enhances the steric exclusion of the dyes. As a result, the selectivity between OR- and NGB3- dropped. Generally, better removal of NGB3- than OR- is observed by the TA-Fe^3+^ membranes owing to both the bigger molecular size and the three ionizable moieties in the NGB3- structure. A comparison of membrane performance for charge-based and size-based solute–solute separation of the TA-Fe^3+^ membranes with those of other studies reported in the literature for small organic molecules is presented in Table 3. A comprehensive comparison with recently published works indicates that our TA-Fe^3+^ membranes exhibit an excellent balance between water flux and solute separation selectivity. This demonstrates that the fabricated membranes hold great potential for improving the separation performances of NF membranes for small organic solutes.

**Table 2:** Separation Selectivity of Solutes in a Mixed Solute Retention Test

membrane	RB0/NGB3- selectivity	OR-/NGB3- selectivity
PAN	1.0	1.0
M1	11.7	3.4
M2	9.8	2.4
M3	18.4	1.8
M4	30.8	1.6

**Table 3:** Performance Comparison of the Fabricated TA-Fe^3+^ Membranes with Other Studies Reported for the Charge- and Size-Based Separation of Small Organic Molecules

membrane type	small organic molecules	molecular weight (g·mol^–1^)	molecular charge	selectivity diffusion^a^	selectivity filtration		water permeance (L·m^–2^·h^–1^·bar^–1^)	ref
					single solutes	mixed solutes		
M1	RB0	376.36	0		8.5	11.7	51	this work
	NGB3-	878.45	–3					
M4	RB0	376.36	0		18.2	30.8	17	this work
	NGB3-	878.45	–3					
M1	RB0	376.36	0		4.5		51	this work
	OR-	350.32	–1					
M4	RB0	376.36	0		8.7		17	this work
	OR-	350.32	–1					
M1	OR-	350.32	–1		1.9	3.4	51	this work
	NGB3-	878.45	–3					
M4	OR-	350.32	–1		2.1	1.6	17	this work
	NGB3-	878.45	–3					
MPN (MPN-1)	RB0	376.36	0		2.0		86.1	(50)
	OR-	350.32	–1					
MPN (MPN-6)	RB0	376.36	0		13.2		5.6	(50)
	OR-	350.32	–1					
MPN (MPN-1)	RB0	376.36	0		5.3	6.5	86.1	(50)
	NGB3-	878.45	–3					
MPN (MPN-6)	RB0	376.36	0		26.7	18.4	5.6	(50)
	NGB3-	878.45	–3					
MPN (MPN-1)	OR-	350.32	–1		2.9	3.6	86.1	(50)
	NGB3-	878.45	–3					
MPN (MPN-6)	OR-	350.32	–1		2	1.9	5.6	(50)
	NGB3-	878.45	–3					
MPN (1TA-3Fe)	RB0	376.36	0		3.2		62.5	(32)
	OR-	350.32	–1					
MPN (1TA-4.5Fe)	RB0	376.36	0		8.5		13.6	(32)
	OR-	350.32	–1					
MPN (1TA-6Fe)	RB0	376.36	0		20.6		3.8	(32)
	OR-	350.32	–1					
MPN (1TA-8Fe)	RB0	376.36	0		3.2		0.9	(32)
	OR-	350.32	–1					
amphiphilic random copolymer membrane	riboflavin	376.36	0	263	8.4^b^	19.2^b^	4.2	(51)
	acid blue 45	474.33	–2					
isoporous positively charged PI-*b*-PS-*b*-P4VP triblock terpolymer membrane (quaternized P4VP block)	RB0	376.36	0		21.3	28.3	11.0	(52)
	methylene blue	319.85	+1					
isoporous negatively charged PI-*b*-PS-*b*-P4VP triblock terpolymer membrane (sulfonated PI block)	OR-	350.32	–1		14.7	44.6	9.5	(52)
	NGB3-	878.45	–3					
isoporous negatively charged PI-*b*-PS-*b*-P4VP triblock terpolymer membrane (sulfonated PI block)	OR-	350.32	–1		64.3		9.5	(52)
	reactive green 19	1418.93	–6					
isoporous positively charged PS-*b*-P4VP diblock copolymer membrane (quaternized P4VP block)	RB0	376.36	0		35.7	39.9	3.8	(53)
	methylene blue	319.85	+1					
isoporous negatively charged PS-*b*-PI diblock copolymer membrane (sulfonated PI block)	OR-	350.32	–1		5.2		74	(54)
	reactive green 19	1418.93	–6					
NP-Den hybrid membrane	rhodamine 6G	479.02	+1	11				(55)
	calcein	622.53	–4					
self-assembled polyelectrolyte deposited PCTE	rhodamine 6G	479.02	+1	3.5				(56)
	calcein	622.53	–4					
cationic dendrimer deposited PCTE	calcein	622.53	–4	10				(57)
	rhodamine 6G	479.02	+1					

a The selectivities were determined from a single-solute system.

b We calculated the selectivities using the reported retention values. RB0, riboflavin; NGB3-, naphthol green B; OR-, orange II; MPN, metal-polyphenol network; MPN-1, 1TA-4.5Fe assembly time 1 min; MPN-6, 1TA-4.5Fe assembly time 6 min; NP-Den hybrid membrane, nanoporous membranes via chemically directed assembly of nanoparticles and dendrimers; PCTE, track etched polycarbonate membrane.

#### 3.3.4 Salt Retention

**Figure 10:**
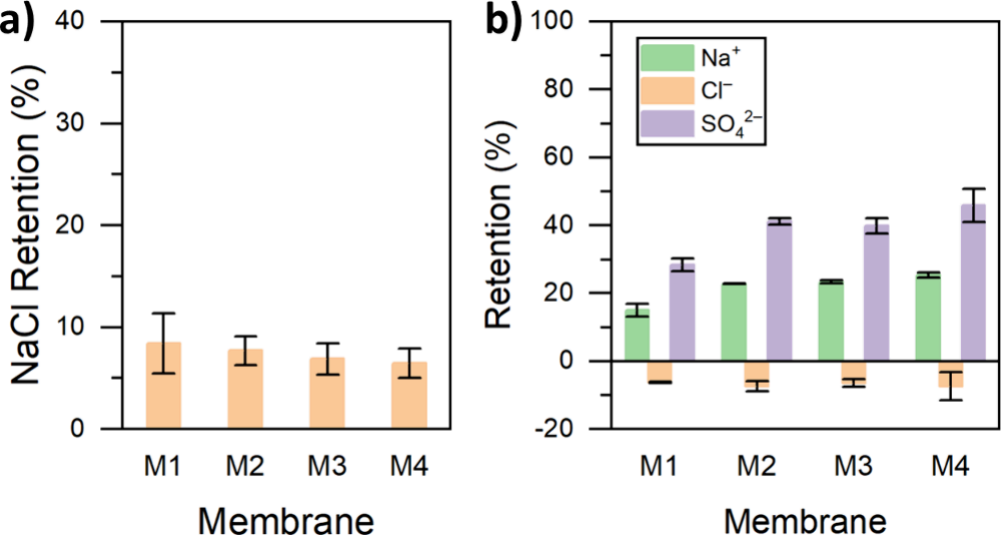
Salt retention performance of the MPN membranes. (a) NaCl single salt solution and (b) NaCl–Na_2_SO_4_ mixed salt solution. Feed solutions were fixed at 1 g/L. A Cl^–^/SO_4_^2–^ molar ratio of 1:1 was used for the mixed salt solution preparation.

The single salt retention behavior of the prepared TFC membranes was determined with NaCl (Figure 10a). The membranes exhibited an extremely low retention of only 6–8% toward NaCl, with no significant impact of the pH at which the membranes were fabricated. The low salt rejection is mainly due to the large pore size of the membranes. The high permeation of salts indicates that the membranes can be beneficial for the separation of dye/salt mixtures since dye wastewater streams contain high concentrations of inorganic salts. To fractionate organic solutes from a saline mixture, salt extraction from the stream by a membrane is a critical issue while also achieving a high retention of the desired organic solute.^58^ Furthermore, low salt retention during organic solute separation prevents an increase in the osmotic pressure of feed solutions, which can significantly reduce the driving force and permeation flux. In order to test the separation performance of the prepared membranes toward mixed ion feed solutions, filtration experiments were performed with a feed solution containing a mixture of NaCl and Na_2_SO_4_. It is obvious from Figure 10b that the retention of chloride by the TFC membranes is significantly lower than that of sulfate. Although the retentions of chloride by the TFC membrane from feed solutions containing only NaCl are slightly positive, in the case of the feed solution containing a mixture of NaCl and Na_2_SO_4_, the retentions of chloride are negative (between −6 and −7%). The sulfate retentions increased from 28 to 46% when the pH of the TA casting solution was increased from 3 (for M1) to 8.5 (for M4). Similarly, Na^+^ retentions slightly improved from 15 to 25%. The higher retention of sulfate compared to that of chloride is a typical characteristic of membranes in the presence of mixed ions of the same charge. The Cl^–^/SO_4_^2–^ selectivity was in the range of 1.5–2, increasing with the pH of the casting solution. This membrane’s selectivity toward the anions can be explained by the larger transport hindrance experienced by the divalent and less diffusive SO_4_^2–^ ion with a larger hydrated size compared to the Cl^–^ ion.^59^ The negative retention of chloride observed in the analyzed salt mixture is a consequence of the electroneutrality condition. This phenomenon has been encountered widely for ternary ion systems in the literature.^60−62^ As Na^+^ is a counterion to the membrane, sodium ions are transported to the permeate side due to the electrochemical potential gradient and a low exclusion by the membrane. Since the divalent and larger SO_4_^2–^ is excluded by the membrane active layer, the more mobile and less charged coion, Cl^–^, diffuses toward the permeate side of the membrane to neutralize the permeate solution. The transported Na^+^ ions pull in the less excluded coion Cl^–^ so that electroneutrality is always fulfilled on both sides of the separation layer. The stirred cell dead-end filtration is performed with a large volume of feed solution compared to that of permeate solutions. Hence, there is a large quantity of chloride ions on the feed side of the membrane compared with the permeate side. The mass transfer of Cl^–^ leads to a higher permeate concentration relative to the feed. This resulted in the observed negative rejection of chloride from the ternary ion mixture.

#### 3.3.5 Antifouling Performance of the TFC Membranes

**Figure 11:**
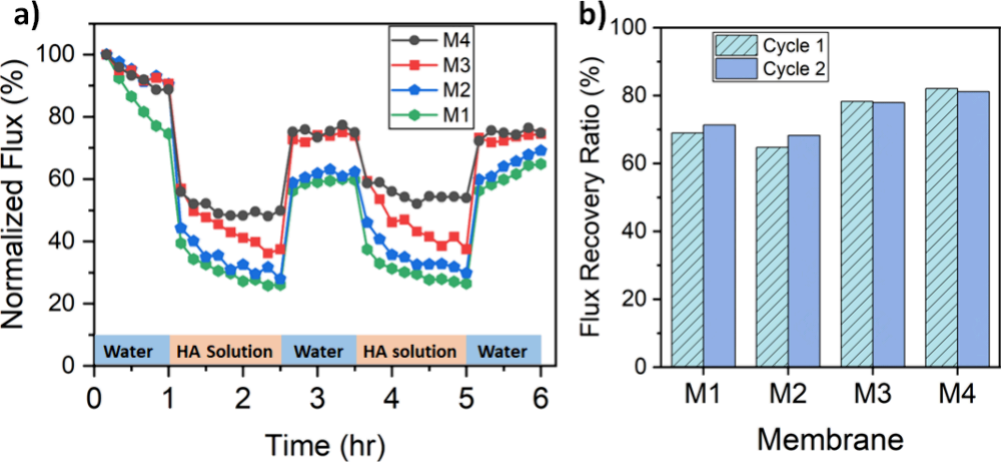
(a) Normalized flux variation during fouling test with HA solution and (b) FRR.

Although membrane development has shown significant progress over the past half-century, membrane fouling remains a key challenge in membrane filtration. The presence of foulant organic compounds such as humic substances^63^ in feed streams can lead to their adsorption on membrane surfaces. High concentrations of humic acid are found in raw wastewater and municipal wastewater effluents. As electrostatic and acid–base interactions strongly govern membrane fouling,^64^ the fabrication of a separation layer with distinct membrane surface chemistry is beneficial in reducing fouling. Fouling studies demonstrate that membrane fouling is affected by hydrodynamic conditions, feedwater characteristics, and membrane properties.^65,66^ The interfacial property of the membrane surface impacts the membrane–foulant interactions. Negatively charged, hydrophilic, and smooth membrane surface properties with weaker Lewis acid–base interactions between the membrane and foulant are beneficial in reducing membrane fouling.^64,65,67^ Here, we investigated the fouling of humic acid on the TA-Fe^3+^ separation layer containing membranes. In order to monitor the performance of the fabricated membranes during the fouling operation, a two-cycle filtration of humic acid solution was measured. Figure 11 shows the normalized flux profiles and the FRRs of the membranes. The water fluxes declined slightly during the pure water filtration in the first 1 h. However, the fluxes decreased significantly when pure water was replaced by the humic acid solution. Comparatively, a small flux decline was observed for the membranes synthesized at high pH. The water flux of the thin films recovered to 69%–82% of their initial value after cleaning. A careful comparison of Figure 11a,b shows that M3 and M4 have higher FRRs compared to M1 and M2. It implies that the lower surface porosity of the TA-Fe^3+^ selective layers coated using the TA casting solutions of pH 5.8 and 8.5 leads to better antifouling properties. It must be taken into account that several factors affect the membrane fouling behavior. Strong electrostatic attractions allow the accumulation of macromolecular foulants on the membrane surface, while electrostatic repulsive forces act contrarily. The TA-Fe^3+^ TFC membrane’s surface chemistry has shown that the membranes exhibit negative zeta potential in the 5–8 pH range (Figure 6a). Humic acid is also negatively charged in the aqueous solution due to the pH-dependent deprotonation of carboxylic and phenolic groups.^68^ Therefore, the electrostatic repulsive force contributes to the overall reduction in the deposition of humic acid on the membrane surface. Second, surface hydrophilicity influences membrane fouling. Hydrophobic membranes have a higher affinity for foulants than those comprising hydrophilic surfaces. Contact angle characterization (Figure 6b) has shown that an increase in hydrophilicity was generally observed in the order of membranes M1–M4. This supports the decrease in the flux decline as well as the increase in flux recovery for the membranes fabricated at high pH. Binding of water molecules to the surface of the hydrophilic thin films through water layer formation prevents membrane fouling.^69^ Moreover, the formation of the hydration layer (water layer) makes the removal of fouled humic acid through cleaning easy.

## 4 Conclusions

The pH of a solution significantly affects metal–polyphenol self-assembly, influencing the structure and performance of the resulting thin film. In this work, we presented the pH of the TA solution as an effective parameter to tailor the membrane surface properties of TA-Fe^3+^ containing TFC membranes fabricated via LBL deposition. In this regard, a series of membranes have been prepared by systematically varying the pH of the TA casting solution for each deposited TA-Fe^3+^ layer or the number of layers deposited, while the other casting parameters were kept constant. The findings of this work showed that TFC membranes with high hydraulic resistance to water transport are fabricated when a TA-Fe^3+^ layer is synthesized at a higher pH on top of an already existing MPN layer synthesized at a low pH. The reverse procedure results in a porous structure with high water permeance. In general, an increase in pH led to a decrease in surface porosity due to the increase in complex formation at a higher pH. For double TA-Fe^3+^ layered TFC membranes where both layers were fabricated at the same pH, increasing the pH of the solution for coating decreased the membrane surface porosity and the water permeation rate while forming more hydrophilic surfaces. The fabricated membranes exhibited low salt retention performance, which can be beneficial for dye/salt fractionation and for reducing the osmotic pressure. In addition, the well-controlled MPN coating showed that neutral organic components PEG and riboflavin can easily permeate through the separation layer, whereas a high retention of anionic dyes by the MPN membranes was achieved. The high selectivities between anionic charged and neutral groups in both single and mixed solute retention reveal that the polyphenol-containing thin films hold great potential for charge-based solute–solute separation. The membranes prepared at different pH values showed excellent performance in separating small organics. The neutral/anionic dye selectivity from mixed dye retention tests increased with an increase in the pH during membrane fabrication. Specifically, the M4 membrane displayed the best riboflavin/naphthol green B separation selectivity of more than 30, the highest selectivity reported for a TFC membrane having an MPN selective layer. The TFC membranes also demonstrated the selective permeation of Cl^–^ ions over SO_4_^2–^ ions from a feed solution containing a Na^+^, SO_4_^2–^, and Cl^–^ ternary ion mixture. Furthermore, it was demonstrated that TA-Fe^3+^ selective layers coated at a higher pH led to a better FRR of the TFC membranes. It is evident that the pH of the TA casting solution is an effective tool for tuning the performance of TFC membranes having TA-Fe^3+^ selective layers.

## Supporting Information Available

FTIR spectra of TA-Fe^3+^ membranes (Figure S1), PWP of MPN TFC membranes (Figures S2 and S3), time-dependent WCA (Figure S4), and selectivity of dyes during single dye retention tests (Table S1) (PDF)
