# Supplementary material for: Nanofiltration Membranes Containing a Metal–Polyphenol Network Layer: Using Casting Solution pH as a Tool to Tailor the Separation Performance
Source: ACS Omega. 2024 Nov 8;9(46):45870–83. doi: 10.1021/acsomega.4c04804 (PMC11579772; doi:10.1021/acsomega.4c04804)
Supplement: Supplementary file 1 — ao4c04804_si_001.pdf [file ao4c04804_si_001.pdf]

Nanofiltration membranes containing a metal-polyphenol network layer: using casting solution pH as a tool to tailor the separation performance

*Hluf Hailu Kinfu[a], Md. Mushfequr Rahman[a, *], Nicolás Cevallos-Cueva[a], and Volker Abetz[a,b]*

[a] Helmholtz-Zentrum Hereon, Institute of Membrane Research, Max-Planck-Straße 1, 21502 Geesthacht, Germany.

[b] University of Hamburg, Institute of Physical Chemistry, Martin-Luther-King-Platz 6, 20146 Hamburg, Germany.

* Corresponding author. E-mail address: mushfequr.rahman@hereon.de

Supporting information

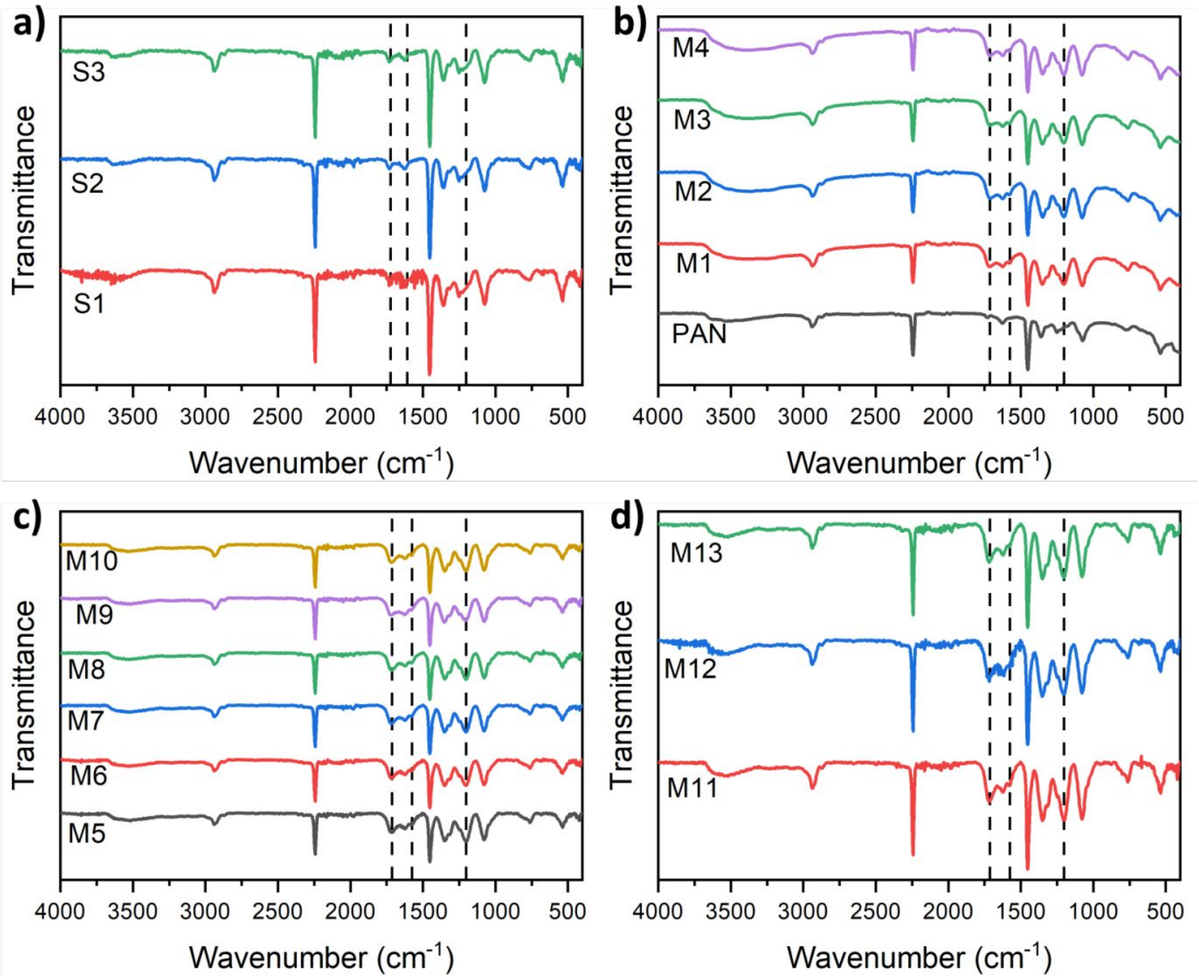

Figure S1. FTIR spectra of a) single TA-Fe^{3+} layered (S1 – S3), b-d) double TA-Fe^{3+} (M1 – M13) layered thin-film composite membranes prepared at various pH of the tannic acid solution.

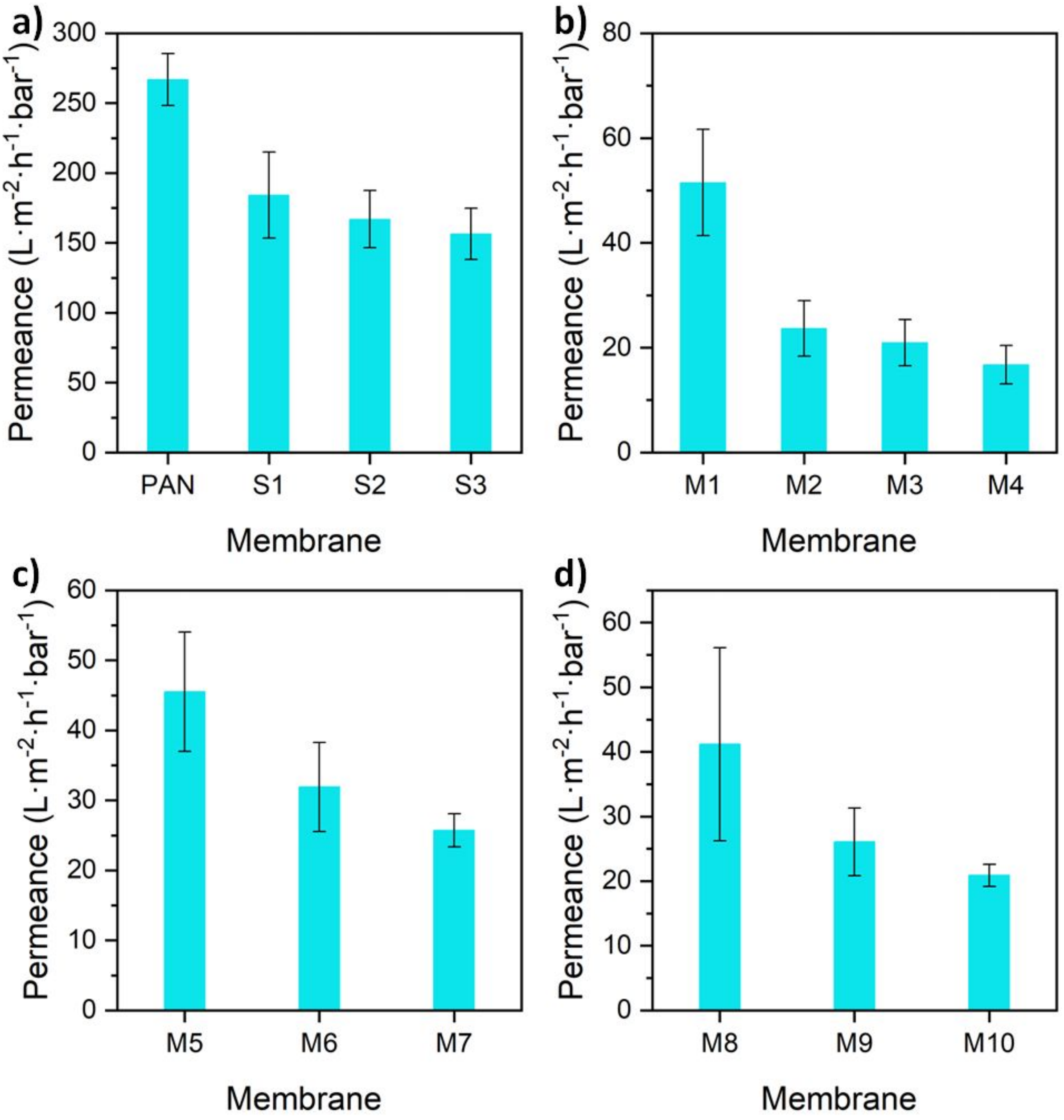

Figure S2. Pure water permeance of MPN TFC membranes: a) single TA-Fe^{3+} coating layer fabricated at different pH of TA solution, b) double TA-Fe^{3+} layered TFC membranes at which both layers were fabricated at the same pH of TA solution, c) double TA-Fe^{3+} layered TFC membranes in which the first layer was synthesized at low pH (pH 3) and the second layer at a higher pH of the TA solution, and d) double TA-Fe^{3+} layered TFC membranes in which the first layer was fabricated at high pH (pH 8.5) while the second layer was at a lower pH. For the complete fabrication conditions, the reader is referred to Table 1 of the main manuscript.

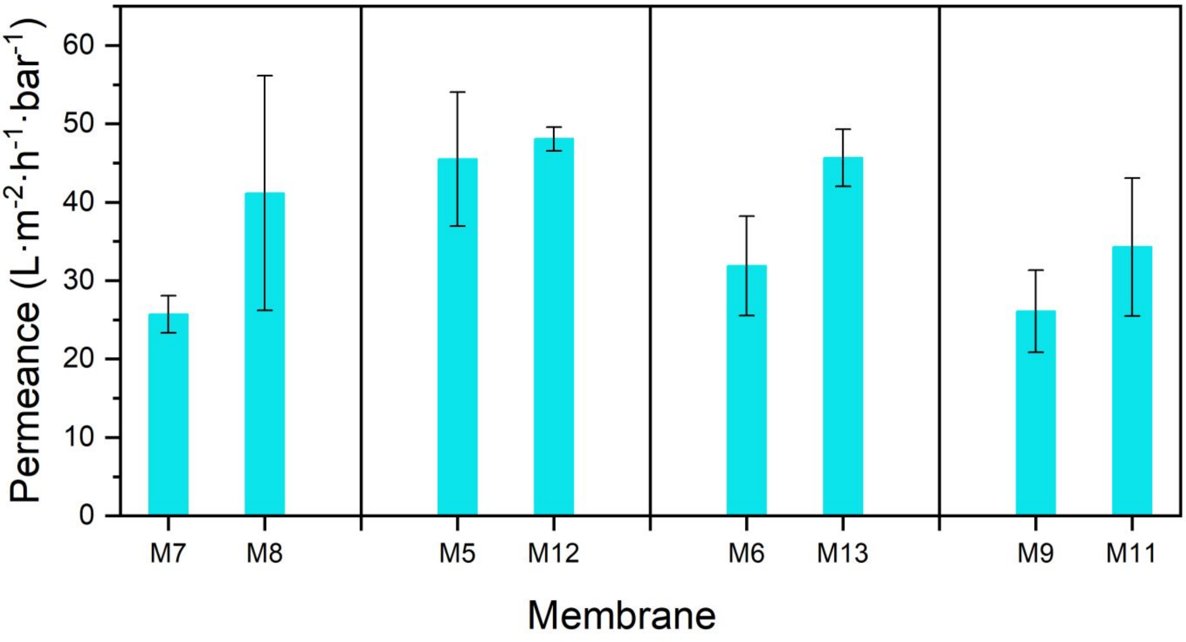

Figure S3. Comparison of pure water permeance of double TA-Fe^{3+} layered TFC membranes fabricated at different pH of TA solution for each layer. The membranes in one column are synthesized by switching the pH condition of each layer. For example, for membrane M7, the first layer is deposited at pH3 and the second layer at pH 8.5. Whereas M8 was fabricated by reversing these steps. For the complete fabrication conditions, the reader is referred to Table 1 of the main manuscript.

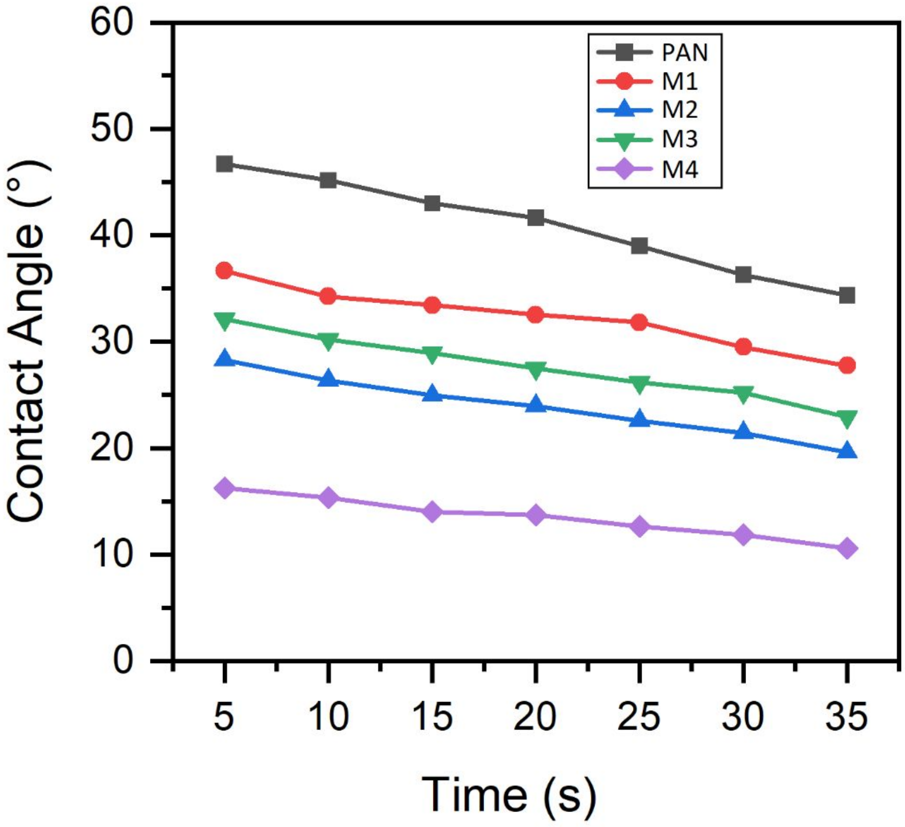

Figure S4. Time dependent variation of the water contact angle for the PAN and TA-Fe^{3+} membranes.

Table S1. Selectivity of solute permeation during single dye measurement by TA-Fe^{3+} membranes fabricated at different TA solution pH.

Ideal selectivities of dye pairs	PAN	M1	M2	M3	M4
Riboflavin to Orange II selectivity	1.0	4.5	5.1	8.5	8.7
Riboflavin to Naphthol green B selectivity	1.0	8.5	11.4	23.4	18.2
Orange II to Naphthol green B selectivity	1.0	1.9	2.2	2.8	2.1
